# Structures of substituted pyridine *N*-oxide with manganese(II) acetate

**DOI:** 10.1107/S205698901801232X

**Published:** 2018-09-11

**Authors:** Will Lynch, Genevieve Lynch, Kirk Sheriff, Clifford Padgett

**Affiliations:** a Department of Chemistry and Biochemistry, Georgia Southern University, 11935 Abercorn Street, Savannah, GA 31419, USA; b St. Vincent’s Academy, Savannah, 31401 Georgia, USA

**Keywords:** crystal structure, manganese(II) acetate, pyridine *N*-oxide ligand, coordination polymer

## Abstract

Manganese(II) acetate coordination polymers have been prepared with three derivatives of pyridine *N*-oxide. The compounds are *catena*-poly[manganese(II)-μ_3_-acetato-di-μ_2_-acetato-[aqua­manganese(II)]-μ_2_-acetato-μ-(pyridine *N*-oxide)-manganese(II)-μ_3_-acetato-μ_2_-acetato-μ-(pyridine *N*-oxide)-[aqua­manganese(II)]-di-μ_2_-acetato], [Mn_4_(CH_3_COO)_8_(C_5_H_5_NO)_2_(H_2_O)_2_]_*n*_, (**I**), *catena*-poly[[manganese(II)]-μ_3_-acetato-μ_2_-acetato-μ-(2-methyl­pyridine *N*-oxide)-[aqua­manganese(II)]-di-μ_2_-acetato-manganese(II)-di-μ_2_-acetato-μ_3_-acetato-[aqua­manganese(II)]-μ_2_-acetato-μ-(2-methyl­pyridine *N*-oxide)], [Mn_4_(CH_3_COO)_8_(C_6_H_7_NO)_2_(H_2_O)_2_]_*n*_, (**II**), and *catena*-poly[[manganese(II)-di-μ_2_-acetato-μ-(4-methyl­pyridine *N*-oxide)] monohydrate], {[Mn(CH_3_COO)_2_(C_6_H_7_NO)]·H_2_O}_*n*_, (**III**). Compounds (**I**) and (**II**) both have three unique Mn atoms; in both compounds two of them sit on a crystallographic inversion center while the third is on a general position. In compound (**III**), the single unique Mn atom sits on a general position. Pseudo-octa­hedral six-coordinate mangan­ese(II) centers are found in all compounds. All of the compounds form chains of Mn atoms bridged by acetate ions and the oxygen atom of the *N-*oxide in pyridine *N-*oxide (PNO), 2-methyl­pyridine *N-*oxide (2MePNO), or 4-methyl­pyridine *N*-oxide (4MePNO). Compound **(I)** and (**II**) both exhibit a bound water of solvation. In (**I**), the water hydrogen bonds to a nearby acetate whereas in (**II**) the water mol­ecule forms bridging hydrogen bonds between two neighboring acetates. In compound **(III)** a water mol­ecule of solvation is found in the lattice, not bound to the metal ion but hydrogen bonding to a bridging acetate.

## Abstract 2

The synthesis and structures of three coordination polymers composed of manganese(II) acetate and pyridine *N*-oxide complexes are reported. The pyridine *N*-oxide, 2-methyl­pyridine *N*-oxide, and 4-methyl­pyridine *N*-oxide complexes form different networks owing to the substituent group effects.

## Chemical context   

*N*-Oxides and acetates both have inter­esting binding modes that facilitate the growth of unique coordination structures. The structures take advantage of the versatility of the acetate ions and the hybridization and dipole at the oxygen atom on the *N*-oxide. The structures extend to the formation of coord­ination polymers that have been reported previously (Sarma *et al.*, 2008 ▸, 2009 ▸; Sarma & Baruah, 2011 ▸). A recent report shows the utility of pyridine *N*-oxide to facilitate coordination polymer formation with both zinc(II) and manganese(II) metal ions with a single bifunctional ligand containing an acetate and *N*-oxide moiety (Ren *et al.*, 2018 ▸). In a previous paper in this series, we examined the initial utility of aromatic *N*-oxide ligands to form polymeric structures with mangan­ese(II) chloride (Kang *et al.*, 2017 ▸). Complexes have also been used previously as metal centers for catalytic transformations (Liu *et al.*, 2014 ▸).
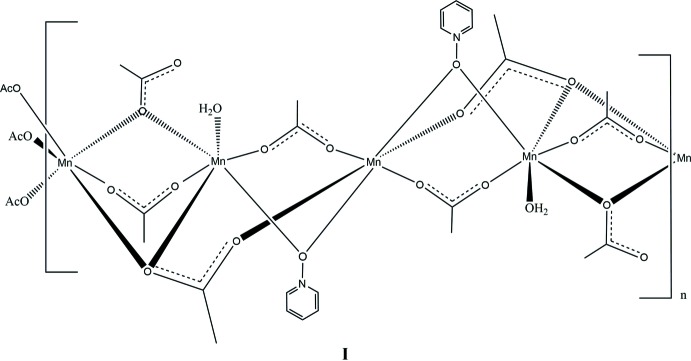
 
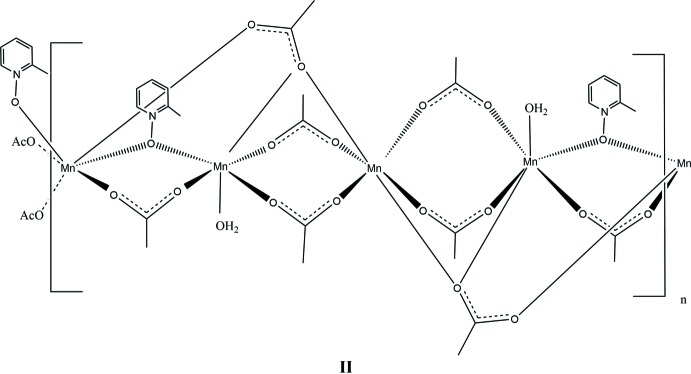
 
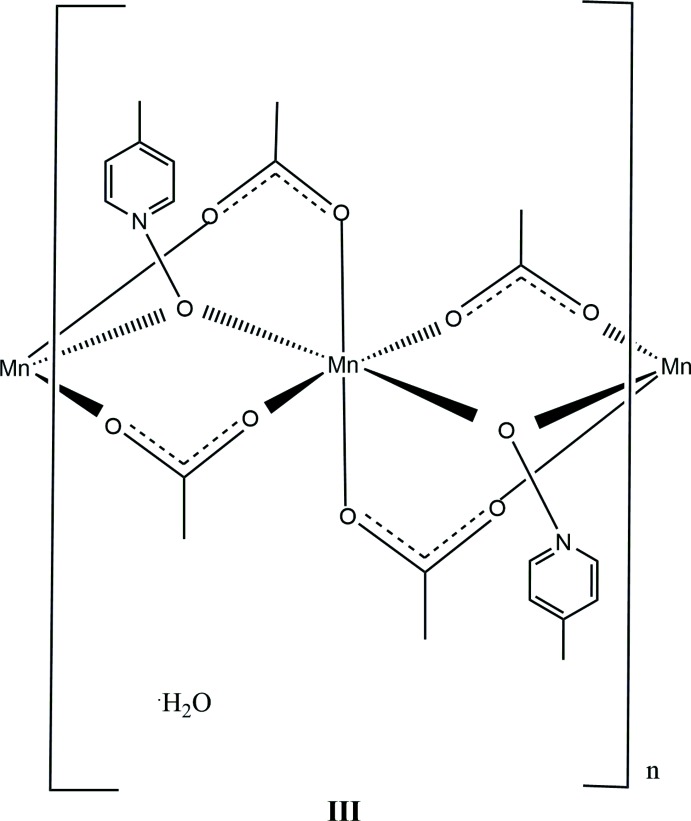


**Figure 1:**
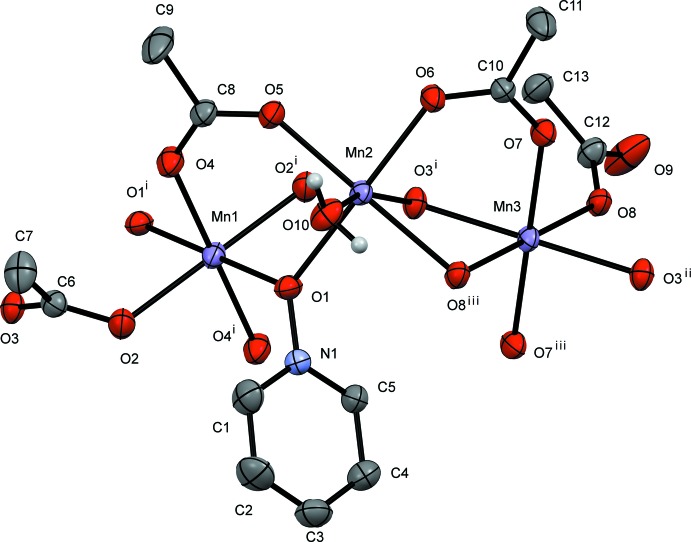
A view of compound **I**, showing the atom labeling. Displacement ellipsoids are drawn at the 50% probability level, H atoms not involved in hydrogen bonding have been omitted for clarity. [Symmetry codes: (i) −*x* + 1, –*y* + 1, −*z* + 1; (ii) *x* + 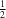, *y* − 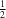, *z*; (iii) −*x* + 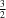, −*y* + 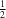, −*z* + 1.]

In this contribution, we report the synthesis and solid-state structures of three manganese(II) complexes with the versatile mono- or bidentate bridging ligands acetate and three deriv­atives of pyridine *N*-oxide (Figs. 1 ▸–3 ▸ ▸). In this study, each of the ligands pyridine *N*-oxide, 2-methyl and 4-methyl pyridine *N*-oxide has an impact on the structures of manganese(II) acetate complexes. All three complexes form coordination polymers with the *N*-oxide bridging in a μ_2_-1,1 mode and varying acetate ligation. The study was conducted to investigate the utility of both acetate and substituted pyridine *N-*oxide to facilitate the growth of unique coordination polymers.

## Structural commentary   

**Figure 4:**
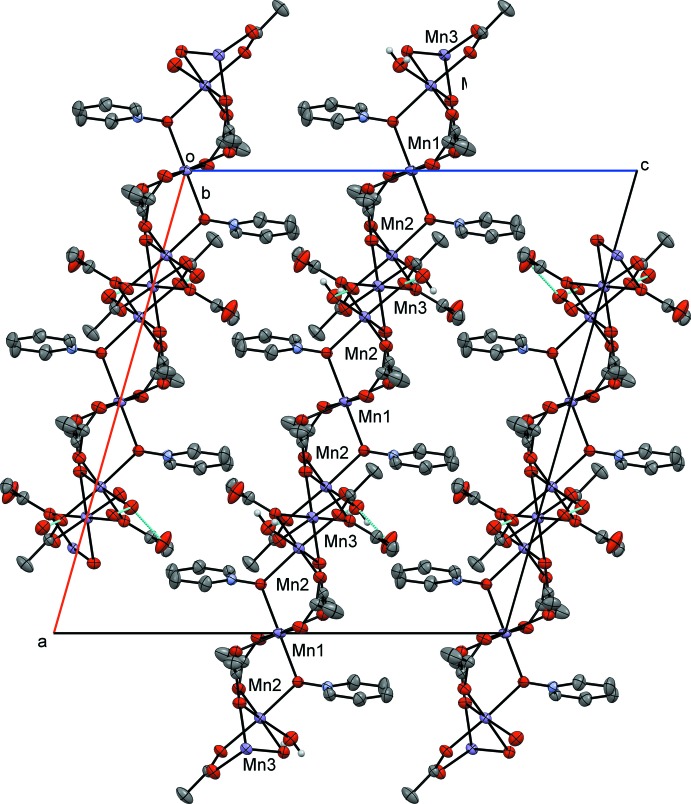
Crystal packing diagram of compound **I**, viewed along the *b* axis. Displacement ellipsoids are drawn at the 50% probability level, H atoms not involved in hydrogen bonding have been omitted for clarity, hydrogen bonds are rendered in blue.

**General structural details.** The pyridine *N*-oxide (PNO) complex, compound **I**, is a repeating tetrameric coordination polymer that crystallizes in the monoclinic space group *C*2/*c*. The manganese atoms align as an Mn3, Mn2, Mn1, Mn2, chain. The structure can be formulated in the simplest empirical relationship as [Mn_4_(PNO)_2_(OAc)_8_(H_2_O)_2_]_*n*_. Examining the mol­ecule across the *AB* vertex, Mn3 and Mn1 sit in a repeating line of Mn3, Mn1, Mn3 atoms. The Mn2 atoms all sit along a different line in this orientation. The atom-to-atom connectivity in the Mn3,Mn2,Mn1,Mn2 repeating unit can best be described as zigzag (Fig. 4 ▸). In this orientation, the pyridine rings also stack; however, they are not π stacked because of the separation distance caused by the methyl group of an acetate ligand in between each aromatic group. Inter­polymeric chain hydrogen bonding is observed from the water mol­ecule (O10) on Mn2 to an oxygen atom (O6) on an Mn2-bound acetate ligand (Table 1 ▸). The structure contains a six-coordinate metal center at each Mn^II^ atom with all six donor atoms being oxygen. Mn1 sits on an inversion center and is bound *trans* by two μ_2_-1,1-PNOs (to Mn2), *trans* by two μ_2_-1,3-acetates (to Mn2), and *trans* by two μ_3_-1,3,3-acetates (to both Mn2 and Mn3). Mn2 is also six-coordinate with a μ_2_-1,1-PNO (from Mn1), a μ_2_-1,3-acetate (from Mn1), and a μ_3_-1,3,3-acetate (from Mn1 and Mn3). Further, the octa­hedral environment is completed by a water of hydration, a μ_2_-1,1-acetate (to Mn3), and a μ_2_-1,3-acetate (to Mn3). Mn3 also sits on an inversion cente, showing an octahedral enviroment where all the six coordinated oxygen atoms belong to acetate ligands. The coordination sphere comprises two μ_3_-1,3,3-acetates (uniquely bound to Mn2 and Mn1), two μ_2_-1,1-acetates (to Mn2) and two μ_2_-1,3-acetates (to Mn2).

**Figure 5:**
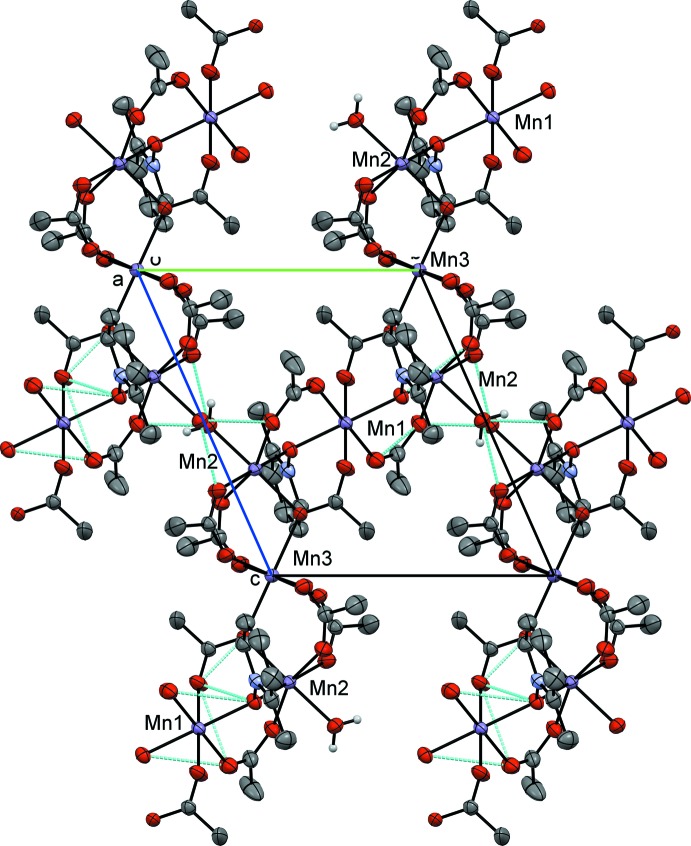
Crystal packing diagram of compound **II**, viewed along the *a* axis. H atoms not involved in hydrogen bonding have been omitted for clarity, hydrogen bonds are rendered in blue.

The 2-methyl­pyridine *N*-oxide (2MePNO) complex, compound **II**, is similar to **I** in that it is a repeating tetrameric coordination polymer. The polymer crystallizes in the triclinic system, space group *P* 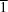. The manganese atoms align as an Mn3, Mn2, Mn1, Mn2 chain similar to **I** with Mn1 and Mn3 sitting on inversion centers. Examining the mol­ecule across the *BC* vertex, as in **I**, the Mn3 and Mn1 sit in a line whereas the Mn2 atoms all sit along a different line with respect to this orientation. The atom-to-atom connectivity in the Mn3, Mn2, Mn1, Mn2 repeating unit can best be described as zigzag (Fig. 5 ▸). In this orientation, the 2-methyl­pyridine ring planes are twisted by 85.31 (2)° with respect to the Mn1/O2/Mn2 plane with all the methyl groups pointing in two symmetry-related directions. As observed in **I**, inter­polymeric chain hydrogen bonding is observed from the water mol­ecule (O10) on Mn2 to an oxygen atom on the adjacent Mn2 on the next polymer. However, symmetry dictates that the hydrogen bonding is to oxygen atoms (O5 and O8) on two acetates bound to Mn2 (Table 2 ▸). The structure can be formulated with the same empirical stoichiometry as **I**, [Mn_4_(2MePNO)_2_(OAc)_7_(H_2_O)_4_]_*n*_. Compound **II** has one important variation from the PNO derivative outlined above. There is no evidence of the μ_2_-1,1-acetate bridge found above. While the singular μ_3_-1,3,3-acetate bridge is retained between Mn2 and Mn3, the μ_2_-1,1 has been replaced by a μ_2_-1,3 acetate bridge. This is likely because of the steric demands of the 2-methyl substituent.

**Figure 6:**
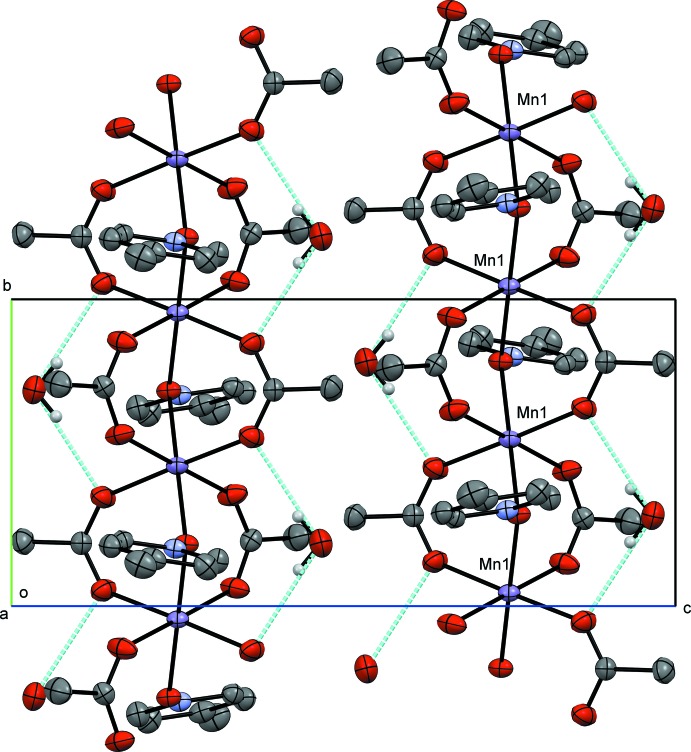
Crystal packing diagram of compound **III**, viewed along the *a* axis. H atoms not involved in hydrogen bonding have been omitted for clarity, hydrogen bonds are rendered in blue.

The 4-methyl­pyridine *N*-oxide (4MePNO) complex, compound **III**, is a repeating coordination polymer with one unique Mn^II^ ion that crystallizes in the monoclinic system, space group *P*2_1_/*n*. In the coordination polymer, the manganese atoms are aligned along the *b-*axis direction. The structure can be formulated as [Mn(4MePNO)_2_(OAc)_4_(H_2_O)]_*n*_. The six-coordinate metal center is bridged by two oxygen atoms from μ_2_-1,1 4MePNO and four μ_2_-1,3 acetate bridges. The 4MePNO complex mol­ecules alternate above and below the line formed by the manganese atoms. Unlike **I** and **II**, compound **III** only forms intra­molecular hydrogen bonding in the polymeric chain (Table 3 ▸, Fig. 6 ▸). The water observed in the lattice forms a hydrogen bond at 2.21 (2) Å with the O2 atom belonging to one of the acetate μ_2_-1,3 acetate bridges.

**Specific structural details.** In **I**, the bond distances involving Mn1 lie between 2.1822 (15) Å (Mn1—O2) and 2.1207 (16) Å (Mn1—O4) whereas all bond angles are within 2.5° of 90°. These angles and distances are similar to those for other Mn^II^ acetate structures (see for example Dave *et al.*, 1993 ▸ and Ciunik & Głowiak, 1980 ▸). The O1 atom of the PNO ligand bridges Mn1 at 2.168 (2) Å and Mn2 at 2.211 (2) Å which is unremarkable for compounds of Mn^II^ and pyridine *N*-oxide (Sniekers *et al.*, 2017 ▸; Mondal *et al.*, 2012 ▸). Mn2 shows a short bond distance to O5 (a μ_2_-1,3-acetate bridging from Mn1) of 2.1062 (15) Å and a long distance of 2.2671 (14) Å from O8, which is a μ_2_-1,1-acetate bridging to Mn3. The water mol­ecule (O10), see Table 1 ▸, is found at 2.1506 (16) Å at a distance similar to that reported previously. (Mondal *et al.*, 2012 ▸) and also hydrogen bonded to O9 (unbound acetate oxygen from μ_2_-1,1-acetate bridging across Mn2 and Mn3) at 2.652 (2) Å. The O3—Mn2—O10 bond angle is severely distorted from 180° to 162.68 (6)°. The other two *trans* bond angles around Mn2 are approximately 175°. The Mn3 bond distances span from 2.1338 (15) (Mn3—O7) to 2.2194 (14) Å (Mn3—O8). The O3—Mn3—O8 bond angle is somewhat constrained at 78.19 (5)° whereas the remaining angles are all nearly 90°.

The bond distances involving Mn1 in compound **II** lie between 2.129 (2) Å (Mn1—O4) and 2.2061 (19) Å (Mn1—O1) which are normal for Mn^II^ acetate compounds of this type (Dave *et al.*, 1993 ▸ and Ciunik & Głowiak, 1980 ▸). The long bond distance is to the oxygen originating from the bridging 2MePNO and is 0.0398 Å longer than the Mn1—O1 PNO bond in **I** but similar to those reported previously (Sniekers *et al.*, 2017 ▸; Mondal *et al.*, 2012 ▸). The bond angles are within 5° of the expected 90° for octa­hedral systems with the most constrained angle being O1—Mn1—O2 [85.03 (7)°]. Mn2 has its shortest bond distance to O6 (a μ_2_-1,3-acetate bridging from Mn3) of 2.136 (2) Å, whereas its longest distance is 2.239 (2) Å to O10, the terminal water mol­ecule. The μ_2_-1,1-2MePNO (O1) bond distance to Mn2 is also long [2.2300 (18) Å]. The μ_3_-1,3,3-acetate also links Mn2 and Mn3 *via* the O3 atom. The O5—Mn2—O10 bond angle is significantly distorted with a value of 81.63 (8)° as is the O6—Mn2—O10 bond angle of 78.61 (8)°. The μ_3_-1,3,3-acetate oxygen (O3) forms a long bond with Mn3 as well, observed at 2.3091 (18) Å. The other Mn3—O bond distances are unremarkable at approximately 2.15 Å. The bond angles around Mn3 are all within 4° of 90° in the six-coordinate Mn3 environment.

In **compound III**, the Mn1—O1 (4MePNO) bond length is the longest of those in this study, with the metal center at 2.203 (3) Å (Sniekers *et al.*, 2017 ▸; Mondal *et al.*, 2012 ▸) whereas the acetate Mn1—O bond distances range from 2.134 (3) Å to 2.179 (2) Å. The bond angles around the metal center are all within 5° of 90°, with the acetate O—Mn1—O angles being slightly larger, whereas the O(acetate)—Mn1—O(4MePNO) angles are slightly compressed. [For similar compounds, see for example Ciunik & Głowiak (1980 ▸) and Dave *et al.* (1993 ▸).] The water is in the lattice and forms a hydrogen bond at 2.21 (2) Å with an O2 atom belonging to one of the acetate μ_2_-1,3 acetate bridges.

## Supra­molecular features   

The packing of **I** forms a polymeric structure bis­ecting the *a* axis and *b* axis. Because of the complexity of the structure, many of the details were outlined above. The structure is not linear but forms a zigzag chain in which the bridg­ing acetates and *N*-oxide ligands connect the Mn ions. There is no evidence for π stacking but interpolymeric chain hydrogen bonding is present.

Compound **II** forms a similar polymeric structure to **I**, with the chain bis­ecting the unit cell at (½, 0, 1) and (½, 1, 0). The chain sets up in a similar fashion as **I;** however, μ_2_-1,3 and μ_3_-1,3,3 are the bridges observed while the μ_2_-1,1 bridge noted in **I** is absent.

Compound **III** forms a polymeric chain which is observed in the *b*-axis direction. Each manganese(II) atom is bridged by a single 4MePNO and two μ_2_-1,3 acetate ions. The 4MePNO bridging ligands are alternating up and down in the *a*-axis direction. There is no evidence for π stacking due to the long distance found in the structure with the aromatic rings at separations of 7.334 (6) Å.

## Database survey   

A search in the Cambridge Structural Database (CSD Version 5.39, November 2017 update; Groom *et al.*, 2016 ▸) for aromatic *N*-oxides and acetate ligands bound to manganese returned 36 entries. Seven of the entries contain derivatives of picolinic *N*-oxides, thirteen involve derivatives of dipyridal *N*-oxide and fifteen include di- or tri-acetate ligands. Similar *N*-oxides with simple benzoate in the list include pyridine *N*-oxide (YIYRAA; Sarma *et al.*, 2008 ▸), and the *p*-nitro­benzoate with PNO (TIXKER01 and TIXKER; Sarma *et al.*, 2008 ▸). Another report by Sarma and co-workers includes the *p*-hy­droxy, *o*-nitro and *p*-chloro­benzoate derivatives with PNO (POYRAX; Sarma *et al.*, 2009 ▸).

## Synthesis and crystallization   

The manganese(II) coordination polymers were all synthesized by a similar method. 0.245 g (1.00 mmol) manganese(II) acetate tetra­hydrate (MnAc_2_·4H_2_O, FW 245 g mol^−1^) was dissolved in a minimal amount (20 mL) of methanol. 2 molar equivalents of the appropriate *N*-oxide (0.191 g pyridine *N*-oxide, PNO; 0.220 g 2-methyl­pyrdine *N*-oxide, 2MePNO; 0.220 g 4-methyl­pyridine *N*-oxide, 4MePNO) were similarly dissolved in 10 mL of methanol. The *N*-oxide alcoholic solution was added in one portion to the Mn^II^ one. The combined reaction mixture was stirred for 30 minutes, filtered and the filtrate was allowed to evaporate by slow diffusion. X-ray quality crystals were obtained by precipitation from the mother liquor. A final wash with a minimal amount of methanol was performed to assist with removal of excess *N*-oxide.

**Compound I**. Yield 0.0642 g, 27.0 (%), decomposition/melting temperature = 433–437 K (turns to a brown liquid). Selected IR bands (ATR, FT–IR, KBr composite, cm^−1^): 3356 (2, *br*), 3118 (*w*), 1558 (*s*), 1495 (*m*), 1477 (*m*), 1418 (*m*), 1424 (*m*), 1216 (*m*), 1025 (*w*), 835 (*m*), 783 (*m*), 653 (*w*).

**Compound II**. Yield 0.0484 g, 20.4 (%), decomposition/melting temperature. 417–423 K (turns to a brown liquid). Selected IR bands (ATR, FT–IR, KBr composite, cm^−1^): 3348 (*m*, *br*), 1558 (*s*), 1495 (*m*), 1417 (*s*), 1209 (*m*), 846 (*m*), 783 (*s*), 655 (*s*).

**Compound III**. Yield 0.0892 g, 29.7 (%), decomposition/melting temperature. 405–411 K (turns to a black liquid). Selected IR bands (ATR, FT–IR, KBr composite, cm^−1^): 3412 (*m*, *br*), 1652 (*w*), 1574 (*s*), 1491 (*s*), 1424 (*m*), 1213 (*s*), 833 (*m*), 763 (*s*), 668 (*s*).

## Refinement   

Crystal data, data collection and structure refinement details are summarized in Table 4 ▸. All carbon-bound H atoms were positioned geometrically and refined as riding, with C—H = 0.95 or 0.98 Å and *U*_iso_(H) = 1.2*U*_eq_(C) or *U*_iso_(H) = 1.5*U*_eq_(C) for C(H) and CH_3_ groups, respectively. In order to ensure chemically meaningful O—H distances for the bound water mol­ecules in the compounds, the oxygen-to-hydrogen distances were restrained to a target value of 0.84 (2) Å (using a DFIX command in *SHELXL2017*; Sheldrick, 2015*b* ▸) and *U*_iso_(H) = 1.5*U*_eq_(O).

## Supplementary Material

Crystal structure: contains datablock(s) I, II, III. DOI: 10.1107/S205698901801232X/zl2728sup1.cif

Structure factors: contains datablock(s) I. DOI: 10.1107/S205698901801232X/zl2728Isup2.hkl

Structure factors: contains datablock(s) II. DOI: 10.1107/S205698901801232X/zl2728IIsup3.hkl

Structure factors: contains datablock(s) III. DOI: 10.1107/S205698901801232X/zl2728IIIsup4.hkl

CCDC references: 1864753, 1864752, 1864751

Additional supporting information:  crystallographic information; 3D view; checkCIF report

## Figures and Tables

**Figure 2:**
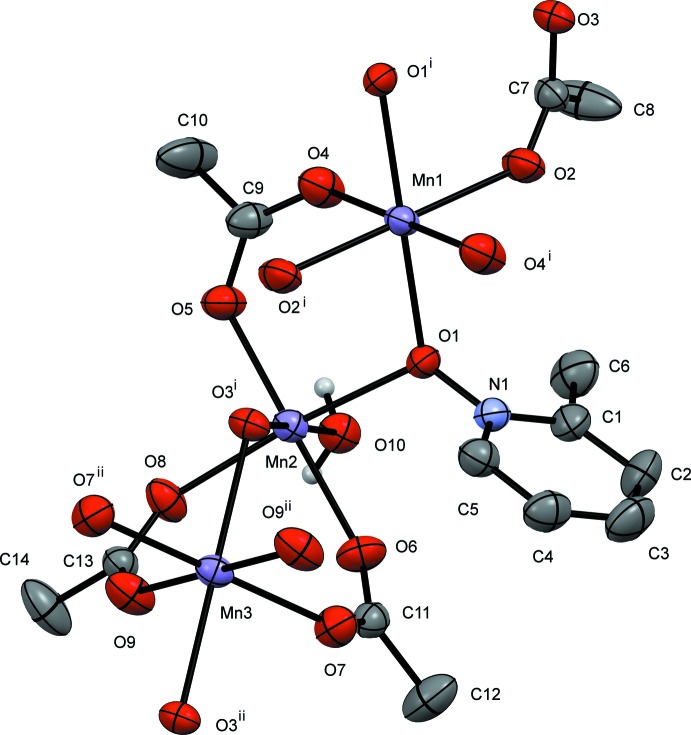
A view of compound **II**, showing the atom labeling. Displacement ellipsoids are drawn at the 50% probability level, H atoms not involved in hydrogen bonding have been omitted for clarity. [Symmetry codes: (i) −*x* + 1, −*y* + 1, −*z* + 1; (ii) −*x* + 1, −*y*, −*z* + 2.]

**Figure 3:**
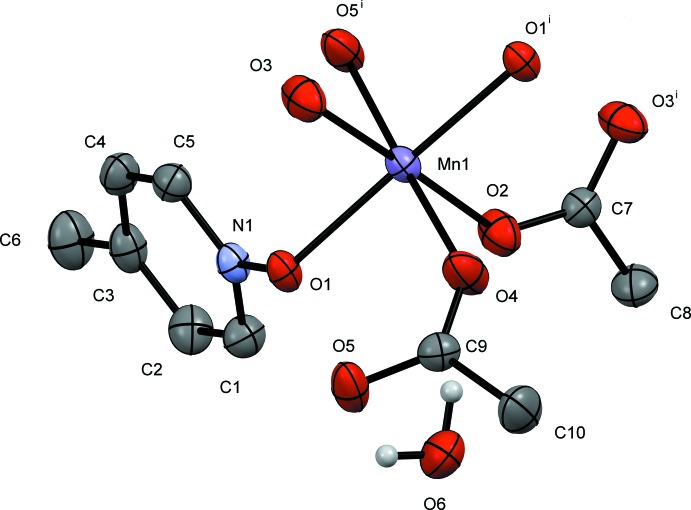
A view of compound **III**, showing the atom labeling. Displacement ellipsoids are drawn at the 50% probability level, H atoms not involved in hydrogen bonding have been omitted for clarity. [Symmetry code: (i) −*x* + 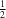, *y* − 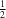, −*z* + 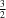.]

**Table 1:** Hydrogen-bond geometry (Å, °) for **I** [Chem scheme1]

*D*—H⋯*A*	*D*—H	H⋯*A*	*D*⋯*A*	*D*—H⋯*A*
O10—H10*A*⋯O9^i^	0.85 (2)	1.85 (2)	2.652 (2)	157 (3)
O10—H10*B*⋯O6^ii^	0.83 (2)	1.98 (2)	2.786 (2)	166 (3)

Symmetry codes: (i) 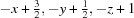; (ii) 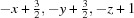.

**Table 2:** Hydrogen-bond geometry (Å, °) for **II** [Chem scheme1]

*D*—H⋯*A*	*D*—H	H⋯*A*	*D*⋯*A*	*D*—H⋯*A*
O10—H10*D*⋯O5^i^	0.84 (2)	2.04 (2)	2.821 (3)	155 (3)
O10—H10*E*⋯O8^i^	0.85 (2)	1.94 (2)	2.727 (3)	155 (3)

Symmetry code: (i) 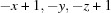.

**Table 3:** Hydrogen-bond geometry (Å, °) for **III** [Chem scheme1]

*D*—H⋯*A*	*D*—H	H⋯*A*	*D*⋯*A*	*D*—H⋯*A*
O6—H6*D*⋯O3^i^	0.84 (2)	2.23 (2)	3.052 (3)	165 (4)
O6—H6*E*⋯O2	0.84 (2)	2.21 (2)	3.035 (3)	170 (4)

Symmetry code: (i) 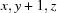.

**Table 4:** Experimental details

	**I**	**II**	**III**
Crystal data			
Chemical formula	[Mn_4_(C_2_H_3_O_2_)_8_(C_5_H_5_NO)_2_(H_2_O)_2_]	[Mn_4_(C_2_H_3_O_2_)_8_(C_6_H_7_NO)_2_(H_2_O)_2_]	[Mn(C_2_H_3_O_2_)_2_(C_6_H_7_NO)]·H_2_O
*M*_r_	918.34	946.40	300.17
Crystal system, space group	Monoclinic, *C*2/*c*	Triclinic, *P* 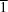	Monoclinic, *P*2_1_/*n*
Temperature (K)	173	173	173
*a*, *b*, *c* (Å)	19.936 (7), 10.603 (4), 18.692 (7)	9.7704 (3), 10.5882 (7), 11.4720 (2)	11.100 (3), 7.334 (3), 15.9808 (4)
α, β, γ (°)	90, 105.925 (4), 90	65.76 (2), 83.84 (2), 65.512 (15)	90, 96.500 (11), 90
*V* (Å^3^)	3800 (2)	982.0 (2)	1292.6 (6)
*Z*	4	1	4
Radiation type	Mo *K*α	Mo *K*α	Mo *K*α
μ (mm^−1^)	1.38	1.34	1.04
Crystal size (mm)	0.4 × 0.4 × 0.2	0.3 × 0.3 × 0.2	0.2 × 0.05 × 0.05

Data collection			
Diffractometer	Rigaku XtaLAB mini	Rigaku XtaLAB mini	Rigaku XtaLAB mini
Absorption correction	Multi-scan (*REQAB*; Rigaku, 1998 ▸)	Multi-scan (*REQAB*; Rigaku, 1998 ▸)	Multi-scan (*REQAB*; Rigaku, 1998 ▸)
*T*_min_, *T*_max_	0.885, 1.00	0.842, 1.00	0.850, 1.00
No. of measured, independent and observed [*I* > 2σ(*I*)] reflections	19620, 4332, 3855	10542, 4503, 3551	13236, 2957, 2224
*R*_int_	0.046	0.058	0.074
(sin θ/λ)_max_ (Å^−1^)	0.649	0.650	0.651

Refinement			
*R*[*F* ^2^ > 2σ(*F* ^2^)], *wR*(*F* ^2^), *S*	0.032, 0.083, 1.08	0.041, 0.101, 1.06	0.045, 0.105, 1.07
No. of reflections	4332	4503	2957
No. of parameters	249	259	173
No. of restraints	2	2	2
H-atom treatment	H atoms treated by a mixture of independent and constrained refinement	H atoms treated by a mixture of independent and constrained refinement	H atoms treated by a mixture of independent and constrained refinement
Δρ_max_, Δρ_min_ (e Å^−3^)	0.31, −0.36	0.62, −0.46	0.35, −0.40

Computer programs: *CrystalClear* (Rigaku, 2009 ▸), *SHELXT* (Sheldrick, 2015*a* ▸), *SHELXL* (Sheldrick, 2015*b* ▸) and *OLEX2* (Dolomanov *et al.*, 2009 ▸).

## supplementary crystallographic information

### catena-Poly[manganese(II)-µ3-acetato-di-µ2-acetato-[aquamanganese(II)]-µ2-acetato-µ-(pyridine N-oxide)-manganese(II)-µ3-acetato-µ2-acetato-µ-(pyridine N-oxide)-[aquamanganese(II)]-di-µ2-acetato] (I) Crystal data

**Table:**

[Mn_4_(C_2_H_3_O_2_)_8_(C_5_H_5_NO)_2_(H_2_O)_2_]	*F*(000) = 1872
*M**_r_* = 918.34	*D*_x_ = 1.605 Mg m^−^^3^
Monoclinic, *C*2/*c*	Mo *K*α radiation, λ = 0.71073 Å
*a* = 19.936 (7) Å	Cell parameters from 5012 reflections
*b* = 10.603 (4) Å	θ = 2.1–27.5°
*c* = 18.692 (7) Å	µ = 1.38 mm^−^^1^
β = 105.925 (4)°	*T* = 173 K
*V* = 3800 (2) Å^3^	Prism, colorless
*Z* = 4	0.4 × 0.4 × 0.2 mm

### catena-Poly[manganese(II)-µ3-acetato-di-µ2-acetato-[aquamanganese(II)]-µ2-acetato-µ-(pyridine N-oxide)-manganese(II)-µ3-acetato-µ2-acetato-µ-(pyridine N-oxide)-[aquamanganese(II)]-di-µ2-acetato] (I) Data collection

**Table:**

Rigaku XtaLAB mini diffractometer	4332 independent reflections
Radiation source: Sealed Tube	3855 reflections with *I* > 2σ(*I*)
Graphite Monochromator monochromator	*R*_int_ = 0.046
Detector resolution: 13.6612 pixels mm^-1^	θ_max_ = 27.5°, θ_min_ = 2.1°
profile data from ω–scans	*h* = −25→25
Absorption correction: multi-scan (*REQAB*; Rigaku, 1998)	*k* = −13→13
*T*_min_ = 0.885, *T*_max_ = 1.00	*l* = −24→24
19620 measured reflections	

### catena-Poly[manganese(II)-µ3-acetato-di-µ2-acetato-[aquamanganese(II)]-µ2-acetato-µ-(pyridine N-oxide)-manganese(II)-µ3-acetato-µ2-acetato-µ-(pyridine N-oxide)-[aquamanganese(II)]-di-µ2-acetato] (I) Refinement

**Table:**

Refinement on *F*^2^	Hydrogen site location: mixed
Least-squares matrix: full	H atoms treated by a mixture of independent and constrained refinement
*R*[*F*^2^ > 2σ(*F*^2^)] = 0.032	*w* = 1/[σ^2^(*F*_o_^2^) + (0.0348*P*)^2^ + 3.1638*P*] where *P* = (*F*_o_^2^ + 2*F*_c_^2^)/3
*wR*(*F*^2^) = 0.083	(Δ/σ)_max_ = 0.001
*S* = 1.08	Δρ_max_ = 0.31 e Å^−^^3^
4332 reflections	Δρ_min_ = −0.36 e Å^−^^3^
249 parameters	Extinction correction: SHELXL-2018/1 (Sheldrick 2015), Fc^*^=kFc[1+0.001xFc^2^λ^3^/sin(2θ)]^-1/4^
2 restraints	Extinction coefficient: 0.00109 (13)
Primary atom site location: dual	

### catena-Poly[manganese(II)-µ3-acetato-di-µ2-acetato-[aquamanganese(II)]-µ2-acetato-µ-(pyridine N-oxide)-manganese(II)-µ3-acetato-µ2-acetato-µ-(pyridine N-oxide)-[aquamanganese(II)]-di-µ2-acetato] (I) Special details

**Table:**

Geometry. All esds (except the esd in the dihedral angle between two l.s. planes) are estimated using the full covariance matrix. The cell esds are taken into account individually in the estimation of esds in distances, angles and torsion angles; correlations between esds in cell parameters are only used when they are defined by crystal symmetry. An approximate (isotropic) treatment of cell esds is used for estimating esds involving l.s. planes.

### catena-Poly[manganese(II)-µ3-acetato-di-µ2-acetato-[aquamanganese(II)]-µ2-acetato-µ-(pyridine N-oxide)-manganese(II)-µ3-acetato-µ2-acetato-µ-(pyridine N-oxide)-[aquamanganese(II)]-di-µ2-acetato] (I) Fractional atomic coordinates and isotropic or equivalent isotropic displacement parameters (Å^2^)

**Table:**

	*x*	*y*	*z*	*U*_iso_*/*U*_eq_	
Mn1	0.500000	0.500000	0.500000	0.02291 (11)	
O1	0.60564 (7)	0.50006 (13)	0.57232 (8)	0.0254 (3)	
C1	0.60680 (13)	0.5206 (2)	0.69567 (13)	0.0368 (5)	
H1	0.594602	0.605051	0.687011	0.044*	
N1	0.61602 (8)	0.44789 (16)	0.64043 (9)	0.0226 (3)	
Mn2	0.68359 (2)	0.53844 (3)	0.51070 (2)	0.01989 (10)	
C2	0.61555 (15)	0.4693 (3)	0.76534 (13)	0.0455 (6)	
H2	0.609182	0.519044	0.803946	0.055*	
O2	0.45997 (7)	0.59052 (14)	0.58476 (8)	0.0290 (3)	
O3	0.34900 (7)	0.64803 (13)	0.54422 (8)	0.0251 (3)	
C3	0.63378 (13)	0.3441 (2)	0.77773 (13)	0.0398 (6)	
H3	0.640102	0.308856	0.824684	0.048*	
Mn3	0.750000	0.250000	0.500000	0.02146 (11)	
C4	0.64253 (12)	0.2717 (2)	0.71959 (13)	0.0355 (5)	
H4	0.654963	0.187201	0.727123	0.043*	
O4	0.51209 (8)	0.68135 (14)	0.45732 (9)	0.0319 (3)	
O5	0.62046 (8)	0.67838 (14)	0.44529 (8)	0.0287 (3)	
C5	0.63271 (11)	0.3254 (2)	0.65014 (12)	0.0280 (4)	
H5	0.637647	0.276949	0.610427	0.034*	
C6	0.41374 (10)	0.67368 (19)	0.57075 (11)	0.0241 (4)	
O6	0.75532 (7)	0.56777 (13)	0.44447 (8)	0.0269 (3)	
C7	0.43584 (13)	0.8095 (2)	0.58470 (15)	0.0402 (6)	
H7A	0.459117	0.836106	0.548603	0.060*	
H7B	0.467028	0.817737	0.633739	0.060*	
H7C	0.395451	0.861292	0.580629	0.060*	
O7	0.79968 (8)	0.37538 (13)	0.44121 (8)	0.0299 (3)	
O8	0.74244 (7)	0.10035 (13)	0.41514 (7)	0.0239 (3)	
C8	0.56142 (11)	0.72708 (19)	0.43642 (12)	0.0268 (4)	
C9	0.54817 (15)	0.8513 (3)	0.39541 (19)	0.0567 (8)	
H9A	0.520255	0.837263	0.345358	0.085*	
H9C	0.591852	0.888472	0.394560	0.085*	
H9B	0.523979	0.907121	0.420264	0.085*	
O9	0.69630 (12)	−0.01467 (16)	0.31454 (10)	0.0552 (6)	
C10	0.79817 (10)	0.49193 (19)	0.42916 (11)	0.0235 (4)	
O10	0.72800 (9)	0.68131 (14)	0.59148 (9)	0.0324 (3)	
H10A	0.7598 (12)	0.645 (3)	0.6246 (13)	0.049*	
H10B	0.7398 (15)	0.7520 (19)	0.5805 (16)	0.049*	
C11	0.85076 (14)	0.5440 (2)	0.39252 (16)	0.0435 (6)	
H11A	0.833091	0.536035	0.339502	0.065*	
H11B	0.893627	0.497746	0.409250	0.065*	
H11C	0.859103	0.631314	0.405432	0.065*	
C12	0.70807 (11)	0.0878 (2)	0.34617 (11)	0.0286 (4)	
C13	0.68183 (13)	0.2058 (2)	0.30226 (12)	0.0362 (5)	
H13A	0.691076	0.277449	0.334898	0.054*	
H13B	0.632510	0.198775	0.279886	0.054*	
H13C	0.705221	0.216242	0.264085	0.054*	

### catena-Poly[manganese(II)-µ3-acetato-di-µ2-acetato-[aquamanganese(II)]-µ2-acetato-µ-(pyridine N-oxide)-manganese(II)-µ3-acetato-µ2-acetato-µ-(pyridine N-oxide)-[aquamanganese(II)]-di-µ2-acetato] (I) Atomic displacement parameters (Å^2^)

**Table:**

	*U*^11^	*U*^22^	*U*^33^	*U*^12^	*U*^13^	*U*^23^
Mn1	0.0195 (2)	0.0200 (2)	0.0295 (2)	0.00260 (15)	0.00725 (17)	0.00445 (17)
O1	0.0230 (7)	0.0277 (8)	0.0258 (7)	0.0009 (6)	0.0073 (5)	0.0085 (6)
C1	0.0477 (14)	0.0283 (12)	0.0336 (12)	0.0043 (10)	0.0098 (10)	−0.0067 (9)
N1	0.0228 (8)	0.0231 (8)	0.0224 (8)	0.0014 (6)	0.0069 (6)	0.0022 (7)
Mn2	0.02113 (16)	0.01489 (16)	0.02513 (17)	0.00127 (10)	0.00883 (12)	0.00102 (11)
C2	0.0627 (17)	0.0480 (15)	0.0268 (12)	0.0038 (13)	0.0141 (11)	−0.0095 (11)
O2	0.0275 (8)	0.0293 (8)	0.0305 (8)	0.0055 (6)	0.0082 (6)	0.0002 (6)
O3	0.0239 (7)	0.0178 (7)	0.0331 (8)	0.0015 (5)	0.0070 (6)	−0.0026 (6)
C3	0.0437 (14)	0.0487 (15)	0.0263 (11)	0.0020 (11)	0.0087 (10)	0.0076 (10)
Mn3	0.0249 (2)	0.0140 (2)	0.0275 (2)	0.00299 (15)	0.01058 (17)	0.00005 (16)
C4	0.0398 (13)	0.0320 (12)	0.0347 (12)	0.0047 (10)	0.0102 (10)	0.0081 (10)
O4	0.0289 (8)	0.0233 (7)	0.0466 (9)	0.0034 (6)	0.0155 (7)	0.0078 (7)
O5	0.0283 (8)	0.0244 (7)	0.0350 (8)	0.0064 (6)	0.0113 (6)	0.0075 (6)
C5	0.0318 (11)	0.0255 (10)	0.0288 (10)	0.0044 (8)	0.0117 (8)	0.0026 (8)
C6	0.0266 (10)	0.0215 (10)	0.0257 (10)	−0.0009 (8)	0.0095 (8)	−0.0011 (8)
O6	0.0292 (8)	0.0204 (7)	0.0361 (8)	0.0031 (6)	0.0173 (6)	0.0035 (6)
C7	0.0323 (12)	0.0270 (12)	0.0611 (16)	−0.0051 (9)	0.0124 (11)	−0.0075 (11)
O7	0.0394 (9)	0.0180 (7)	0.0390 (8)	0.0044 (6)	0.0218 (7)	0.0033 (6)
O8	0.0282 (7)	0.0188 (7)	0.0248 (7)	0.0045 (5)	0.0072 (6)	0.0000 (5)
C8	0.0285 (11)	0.0204 (10)	0.0325 (11)	0.0026 (8)	0.0101 (8)	0.0039 (8)
C9	0.0467 (16)	0.0354 (14)	0.096 (2)	0.0137 (12)	0.0331 (15)	0.0358 (15)
O9	0.0897 (16)	0.0254 (9)	0.0329 (9)	0.0061 (9)	−0.0127 (9)	−0.0053 (7)
C10	0.0256 (10)	0.0221 (10)	0.0251 (10)	0.0022 (8)	0.0107 (8)	0.0017 (8)
O10	0.0397 (9)	0.0176 (7)	0.0364 (9)	−0.0023 (6)	0.0045 (7)	−0.0003 (6)
C11	0.0506 (15)	0.0286 (12)	0.0652 (17)	0.0046 (10)	0.0394 (13)	0.0088 (11)
C12	0.0334 (11)	0.0239 (10)	0.0262 (10)	0.0025 (8)	0.0042 (8)	−0.0014 (8)
C13	0.0454 (13)	0.0314 (12)	0.0294 (11)	0.0092 (10)	0.0062 (10)	0.0042 (9)

### catena-Poly[manganese(II)-µ3-acetato-di-µ2-acetato-[aquamanganese(II)]-µ2-acetato-µ-(pyridine N-oxide)-manganese(II)-µ3-acetato-µ2-acetato-µ-(pyridine N-oxide)-[aquamanganese(II)]-di-µ2-acetato] (I) Geometric parameters (Å, º)

**Table:**

Mn1—O1^i^	2.1680 (15)	C4—H4	0.9300
Mn1—O1	2.1681 (15)	C4—C5	1.381 (3)
Mn1—O2^i^	2.1822 (15)	O4—C8	1.251 (3)
Mn1—O2	2.1822 (15)	O5—C8	1.254 (2)
Mn1—O4^i^	2.1207 (16)	C5—H5	0.9300
Mn1—O4	2.1207 (16)	C6—C7	1.508 (3)
O1—N1	1.351 (2)	O6—C10	1.262 (2)
O1—Mn2	2.2113 (15)	C7—H7A	0.9600
C1—H1	0.9300	C7—H7B	0.9600
C1—N1	1.341 (3)	C7—H7C	0.9600
C1—C2	1.377 (3)	O7—C10	1.255 (2)
N1—C5	1.341 (3)	O8—C12	1.290 (2)
Mn2—O3^i^	2.2404 (15)	C8—C9	1.510 (3)
Mn2—O5	2.1062 (15)	C9—H9A	0.9600
Mn2—O6	2.1551 (15)	C9—H9C	0.9600
Mn2—O8^ii^	2.2671 (14)	C9—H9B	0.9600
Mn2—O10	2.1506 (16)	O9—C12	1.228 (3)
C2—H2	0.9300	C10—C11	1.506 (3)
C2—C3	1.380 (4)	O10—H10A	0.848 (17)
O2—C6	1.250 (2)	O10—H10B	0.829 (17)
O3—Mn3^iii^	2.2028 (15)	C11—H11A	0.9600
O3—C6	1.278 (2)	C11—H11B	0.9600
C3—H3	0.9300	C11—H11C	0.9600
C3—C4	1.380 (3)	C12—C13	1.509 (3)
Mn3—O7^ii^	2.1338 (15)	C13—H13A	0.9600
Mn3—O7	2.1338 (15)	C13—H13B	0.9600
Mn3—O8	2.2194 (14)	C13—H13C	0.9600
Mn3—O8^ii^	2.2194 (14)		
			
O1^i^—Mn1—O1	180.00 (7)	O7—Mn3—O7^ii^	180.0
O1^i^—Mn1—O2^i^	91.91 (6)	O7—Mn3—O8	91.58 (6)
O1—Mn1—O2^i^	88.09 (6)	O7—Mn3—O8^ii^	88.43 (6)
O1^i^—Mn1—O2	88.09 (6)	O7^ii^—Mn3—O8^ii^	91.57 (6)
O1—Mn1—O2	91.91 (6)	O7^ii^—Mn3—O8	88.43 (6)
O2—Mn1—O2^i^	180.0	O8—Mn3—O8^ii^	180.00 (5)
O4^i^—Mn1—O1^i^	92.47 (6)	C3—C4—H4	120.2
O4—Mn1—O1	92.47 (6)	C3—C4—C5	119.7 (2)
O4^i^—Mn1—O1	87.53 (6)	C5—C4—H4	120.2
O4—Mn1—O1^i^	87.53 (6)	C8—O4—Mn1	130.47 (13)
O4^i^—Mn1—O2	91.36 (6)	C8—O5—Mn2	139.17 (14)
O4—Mn1—O2	88.64 (6)	N1—C5—C4	119.5 (2)
O4—Mn1—O2^i^	91.36 (6)	N1—C5—H5	120.2
O4^i^—Mn1—O2^i^	88.64 (6)	C4—C5—H5	120.2
O4—Mn1—O4^i^	180.0	O2—C6—O3	122.61 (19)
Mn1—O1—Mn2	112.12 (6)	O2—C6—C7	118.29 (19)
N1—O1—Mn1	117.31 (11)	O3—C6—C7	119.09 (18)
N1—O1—Mn2	128.21 (11)	C10—O6—Mn2	129.45 (13)
N1—C1—H1	120.3	C6—C7—H7A	109.5
N1—C1—C2	119.5 (2)	C6—C7—H7B	109.5
C2—C1—H1	120.3	C6—C7—H7C	109.5
C1—N1—O1	118.17 (18)	H7A—C7—H7B	109.5
C5—N1—O1	119.51 (16)	H7A—C7—H7C	109.5
C5—N1—C1	122.28 (19)	H7B—C7—H7C	109.5
O1—Mn2—O3^i^	85.37 (6)	C10—O7—Mn3	135.59 (13)
O1—Mn2—O8^ii^	89.66 (5)	Mn3—O8—Mn2^ii^	97.00 (6)
O3^i^—Mn2—O8^ii^	76.44 (5)	C12—O8—Mn2^ii^	128.45 (13)
O5—Mn2—O1	92.21 (6)	C12—O8—Mn3	134.54 (13)
O5—Mn2—O3^i^	107.68 (6)	O4—C8—O5	126.06 (19)
O5—Mn2—O6	87.13 (6)	O4—C8—C9	116.96 (19)
O5—Mn2—O8^ii^	175.59 (6)	O5—C8—C9	116.97 (19)
O5—Mn2—O10	88.66 (6)	C8—C9—H9A	109.5
O6—Mn2—O1	176.02 (6)	C8—C9—H9C	109.5
O6—Mn2—O3^i^	91.08 (6)	C8—C9—H9B	109.5
O6—Mn2—O8^ii^	91.27 (6)	H9A—C9—H9C	109.5
O10—Mn2—O1	88.66 (6)	H9A—C9—H9B	109.5
O10—Mn2—O3^i^	162.77 (6)	H9C—C9—H9B	109.5
O10—Mn2—O6	95.25 (6)	O6—C10—C11	117.96 (18)
O10—Mn2—O8^ii^	87.39 (6)	O7—C10—O6	124.82 (19)
C1—C2—H2	120.1	O7—C10—C11	117.21 (18)
C1—C2—C3	119.9 (2)	Mn2—O10—H10A	106 (2)
C3—C2—H2	120.1	Mn2—O10—H10B	124 (2)
C6—O2—Mn1	123.62 (13)	H10A—O10—H10B	113 (3)
Mn3^iii^—O3—Mn2^i^	98.27 (6)	C10—C11—H11A	109.5
C6—O3—Mn2^i^	120.04 (12)	C10—C11—H11B	109.5
C6—O3—Mn3^iii^	138.28 (13)	C10—C11—H11C	109.5
C2—C3—H3	120.4	H11A—C11—H11B	109.5
C2—C3—C4	119.2 (2)	H11A—C11—H11C	109.5
C4—C3—H3	120.4	H11B—C11—H11C	109.5
O3^i^—Mn3—O3^iv^	180.00 (7)	O8—C12—C13	117.86 (18)
O3^i^—Mn3—O8^ii^	78.19 (5)	O9—C12—O8	123.5 (2)
O3^iv^—Mn3—O8^ii^	101.81 (5)	O9—C12—C13	118.66 (19)
O3^iv^—Mn3—O8	78.19 (5)	C12—C13—H13A	109.5
O3^i^—Mn3—O8	101.81 (5)	C12—C13—H13B	109.5
O7—Mn3—O3^i^	89.75 (6)	C12—C13—H13C	109.5
O7^ii^—Mn3—O3^iv^	89.75 (6)	H13A—C13—H13B	109.5
O7^ii^—Mn3—O3^i^	90.25 (6)	H13A—C13—H13C	109.5
O7—Mn3—O3^iv^	90.25 (6)	H13B—C13—H13C	109.5

Symmetry codes: (i) −*x*+1, −*y*+1, −*z*+1; (ii) −*x*+3/2, −*y*+1/2, −*z*+1; (iii) *x*−1/2, *y*+1/2, *z*; (iv) *x*+1/2, *y*−1/2, *z*.

### catena-Poly[manganese(II)-µ3-acetato-di-µ2-acetato-[aquamanganese(II)]-µ2-acetato-µ-(pyridine N-oxide)-manganese(II)-µ3-acetato-µ2-acetato-µ-(pyridine N-oxide)-[aquamanganese(II)]-di-µ2-acetato] (I) Hydrogen-bond geometry (Å, º)

**Table:**

*D*—H···*A*	*D*—H	H···*A*	*D*···*A*	*D*—H···*A*
O10—H10*A*···O9^ii^	0.85 (2)	1.85 (2)	2.652 (2)	157 (3)
O10—H10*B*···O6^v^	0.83 (2)	1.98 (2)	2.786 (2)	166 (3)

Symmetry codes: (ii) −*x*+3/2, −*y*+1/2, −*z*+1; (v) −*x*+3/2, −*y*+3/2, −*z*+1.

### catena-Poly[[manganese(II)]-µ3-acetato-µ2-acetato-µ-(2-methylpyridine N-oxide)-[aquamanganese(II)]-di-µ2-acetato-manganese(II)-di-µ2-acetato-µ3-acetato-[aquamanganese(II)]-µ2-acetato-µ-(2-methylpyridine N-oxide)] (II) Crystal data

**Table:**

[Mn_4_(C_2_H_3_O_2_)_8_(C_6_H_7_NO)_2_(H_2_O)_2_]	*Z* = 1
*M**_r_* = 946.4	*F*(000) = 484
Triclinic, *P*1	*D*_x_ = 1.600 Mg m^−^^3^
*a* = 9.7704 (3) Å	Mo *K*α radiation, λ = 0.71073 Å
*b* = 10.5882 (7) Å	Cell parameters from 2512 reflections
*c* = 11.4720 (2) Å	θ = 2.0–27.5°
α = 65.76 (2)°	µ = 1.34 mm^−^^1^
β = 83.84 (2)°	*T* = 173 K
γ = 65.512 (15)°	Prism, colorless
*V* = 982.0 (2) Å^3^	0.3 × 0.3 × 0.2 mm

### catena-Poly[[manganese(II)]-µ3-acetato-µ2-acetato-µ-(2-methylpyridine N-oxide)-[aquamanganese(II)]-di-µ2-acetato-manganese(II)-di-µ2-acetato-µ3-acetato-[aquamanganese(II)]-µ2-acetato-µ-(2-methylpyridine N-oxide)] (II) Data collection

**Table:**

Rigaku XtaLAB mini diffractometer	4503 independent reflections
Radiation source: Sealed Tube	3551 reflections with *I* > 2σ(*I*)
Graphite Monochromator monochromator	*R*_int_ = 0.058
Detector resolution: 13.6612 pixels mm^-1^	θ_max_ = 27.5°, θ_min_ = 2.3°
profile data from ω–scans	*h* = −12→12
Absorption correction: multi-scan (*REQAB*; Rigaku, 1998)	*k* = −13→13
*T*_min_ = 0.842, *T*_max_ = 1.00	*l* = −14→14
10542 measured reflections	

### catena-Poly[[manganese(II)]-µ3-acetato-µ2-acetato-µ-(2-methylpyridine N-oxide)-[aquamanganese(II)]-di-µ2-acetato-manganese(II)-di-µ2-acetato-µ3-acetato-[aquamanganese(II)]-µ2-acetato-µ-(2-methylpyridine N-oxide)] (II) Refinement

**Table:**

Refinement on *F*^2^	Hydrogen site location: mixed
Least-squares matrix: full	H atoms treated by a mixture of independent and constrained refinement
*R*[*F*^2^ > 2σ(*F*^2^)] = 0.041	*w* = 1/[σ^2^(*F*_o_^2^) + (0.0271*P*)^2^ + 0.5206*P*] where *P* = (*F*_o_^2^ + 2*F*_c_^2^)/3
*wR*(*F*^2^) = 0.101	(Δ/σ)_max_ < 0.001
*S* = 1.06	Δρ_max_ = 0.62 e Å^−^^3^
4503 reflections	Δρ_min_ = −0.46 e Å^−^^3^
259 parameters	Extinction correction: SHELXL-2018/1 (Sheldrick 2015), Fc^*^=kFc[1+0.001xFc^2^λ^3^/sin(2θ)]^-1/4^
2 restraints	Extinction coefficient: 0.0045 (10)
Primary atom site location: dual	

### catena-Poly[[manganese(II)]-µ3-acetato-µ2-acetato-µ-(2-methylpyridine N-oxide)-[aquamanganese(II)]-di-µ2-acetato-manganese(II)-di-µ2-acetato-µ3-acetato-[aquamanganese(II)]-µ2-acetato-µ-(2-methylpyridine N-oxide)] (II) Special details

**Table:**

Geometry. All esds (except the esd in the dihedral angle between two l.s. planes) are estimated using the full covariance matrix. The cell esds are taken into account individually in the estimation of esds in distances, angles and torsion angles; correlations between esds in cell parameters are only used when they are defined by crystal symmetry. An approximate (isotropic) treatment of cell esds is used for estimating esds involving l.s. planes.

### catena-Poly[[manganese(II)]-µ3-acetato-µ2-acetato-µ-(2-methylpyridine N-oxide)-[aquamanganese(II)]-di-µ2-acetato-manganese(II)-di-µ2-acetato-µ3-acetato-[aquamanganese(II)]-µ2-acetato-µ-(2-methylpyridine N-oxide)] (II) Fractional atomic coordinates and isotropic or equivalent isotropic displacement parameters (Å^2^)

**Table:**

	*x*	*y*	*z*	*U*_iso_*/*U*_eq_	
Mn1	0.500000	0.500000	0.500000	0.02633 (15)	
C1	0.9223 (3)	0.1967 (3)	0.5954 (3)	0.0385 (7)	
O1	0.66323 (19)	0.2610 (2)	0.58767 (17)	0.0294 (4)	
N1	0.7920 (3)	0.2213 (2)	0.6544 (2)	0.0306 (5)	
C2	1.0513 (4)	0.1617 (4)	0.6641 (4)	0.0526 (9)	
H2	1.143547	0.147895	0.624004	0.063*	
O2	0.6422 (2)	0.5705 (2)	0.35280 (18)	0.0405 (5)	
Mn2	0.54423 (4)	0.10659 (4)	0.65107 (4)	0.02574 (12)	
C3	1.0472 (4)	0.1465 (4)	0.7900 (4)	0.0559 (9)	
H3	1.135892	0.121710	0.836731	0.067*	
O3	0.5590 (2)	0.80401 (19)	0.20131 (16)	0.0292 (4)	
Mn3	0.500000	0.000000	1.000000	0.02716 (15)	
C4	0.9121 (4)	0.1680 (4)	0.8465 (3)	0.0446 (8)	
H4	0.907481	0.155806	0.933430	0.053*	
O4	0.3972 (3)	0.4521 (2)	0.37791 (19)	0.0434 (5)	
O5	0.3654 (2)	0.2403 (2)	0.49693 (19)	0.0389 (5)	
C5	0.7842 (4)	0.2072 (3)	0.7767 (3)	0.0375 (7)	
H5	0.690486	0.224241	0.814678	0.045*	
C6	0.9159 (4)	0.2095 (4)	0.4616 (3)	0.0518 (9)	
H6A	0.866513	0.147157	0.458109	0.078*	
H6B	1.018458	0.174165	0.434390	0.078*	
H6C	0.858321	0.315728	0.404389	0.078*	
O6	0.7494 (2)	−0.0675 (2)	0.76008 (18)	0.0411 (5)	
C7	0.6334 (3)	0.6596 (3)	0.2404 (2)	0.0292 (6)	
O7	0.7340 (2)	−0.0951 (2)	0.96318 (18)	0.0391 (5)	
O8	0.4469 (2)	−0.0610 (2)	0.72192 (18)	0.0381 (5)	
C8	0.7129 (5)	0.5940 (4)	0.1464 (3)	0.0695 (13)	
H8A	0.651688	0.650930	0.064547	0.104*	
H8C	0.728199	0.487456	0.179635	0.104*	
H8B	0.810839	0.600750	0.133554	0.104*	
O9	0.4385 (3)	−0.1325 (2)	0.93300 (18)	0.0417 (5)	
C9	0.3347 (4)	0.3642 (3)	0.4000 (3)	0.0388 (7)	
C10	0.2131 (5)	0.4060 (4)	0.3031 (4)	0.0752 (13)	
H10A	0.227713	0.316316	0.288714	0.113*	
H10B	0.218744	0.485502	0.222086	0.113*	
H10C	0.113896	0.443323	0.335646	0.113*	
O10	0.6635 (2)	0.0116 (2)	0.50856 (19)	0.0347 (5)	
H10D	0.669 (4)	−0.076 (2)	0.529 (3)	0.052*	
H10E	0.615 (4)	0.054 (4)	0.436 (2)	0.052*	
C11	0.8010 (3)	−0.1286 (3)	0.8738 (3)	0.0324 (6)	
C12	0.9586 (4)	−0.2532 (5)	0.9039 (3)	0.0574 (10)	
H12B	1.028728	−0.212929	0.850872	0.086*	
H12C	0.959934	−0.336348	0.885672	0.086*	
H12A	0.989418	−0.291080	0.994697	0.086*	
C13	0.4250 (3)	−0.1437 (3)	0.8312 (3)	0.0299 (6)	
C14	0.3769 (5)	−0.2656 (4)	0.8398 (3)	0.0583 (10)	
H14A	0.291205	−0.220931	0.777399	0.087*	
H14C	0.347239	−0.310285	0.926417	0.087*	
H14B	0.461132	−0.344649	0.821308	0.087*	

### catena-Poly[[manganese(II)]-µ3-acetato-µ2-acetato-µ-(2-methylpyridine N-oxide)-[aquamanganese(II)]-di-µ2-acetato-manganese(II)-di-µ2-acetato-µ3-acetato-[aquamanganese(II)]-µ2-acetato-µ-(2-methylpyridine N-oxide)] (II) Atomic displacement parameters (Å^2^)

**Table:**

	*U*^11^	*U*^22^	*U*^33^	*U*^12^	*U*^13^	*U*^23^
Mn1	0.0309 (3)	0.0219 (3)	0.0235 (3)	−0.0121 (2)	−0.0010 (2)	−0.0047 (2)
C1	0.0297 (15)	0.0323 (15)	0.0463 (17)	−0.0089 (13)	0.0010 (13)	−0.0126 (14)
O1	0.0254 (9)	0.0257 (9)	0.0335 (10)	−0.0095 (8)	−0.0038 (8)	−0.0082 (8)
N1	0.0301 (12)	0.0259 (11)	0.0320 (12)	−0.0113 (10)	−0.0046 (10)	−0.0066 (10)
C2	0.0262 (15)	0.058 (2)	0.064 (2)	−0.0109 (15)	−0.0015 (15)	−0.0215 (19)
O2	0.0388 (11)	0.0326 (11)	0.0319 (11)	−0.0104 (9)	0.0061 (9)	−0.0015 (9)
Mn2	0.0309 (2)	0.0255 (2)	0.0225 (2)	−0.01314 (18)	0.00076 (16)	−0.00934 (17)
C3	0.0438 (19)	0.054 (2)	0.065 (2)	−0.0127 (17)	−0.0211 (17)	−0.0211 (19)
O3	0.0372 (10)	0.0228 (9)	0.0237 (9)	−0.0106 (8)	0.0045 (8)	−0.0081 (8)
Mn3	0.0340 (3)	0.0254 (3)	0.0215 (3)	−0.0123 (2)	0.0022 (2)	−0.0089 (2)
C4	0.0488 (19)	0.0428 (18)	0.0398 (17)	−0.0160 (16)	−0.0093 (15)	−0.0144 (15)
O4	0.0600 (14)	0.0399 (12)	0.0350 (11)	−0.0272 (11)	−0.0071 (10)	−0.0100 (10)
O5	0.0449 (12)	0.0278 (10)	0.0385 (11)	−0.0133 (9)	−0.0110 (9)	−0.0065 (9)
C5	0.0430 (17)	0.0346 (16)	0.0351 (15)	−0.0177 (14)	0.0004 (13)	−0.0118 (13)
C6	0.0410 (18)	0.060 (2)	0.0445 (19)	−0.0117 (17)	0.0085 (15)	−0.0222 (18)
O6	0.0430 (12)	0.0371 (11)	0.0324 (11)	−0.0075 (10)	−0.0079 (9)	−0.0105 (9)
C7	0.0300 (14)	0.0264 (13)	0.0290 (14)	−0.0124 (11)	0.0060 (11)	−0.0092 (11)
O7	0.0366 (11)	0.0430 (12)	0.0334 (11)	−0.0120 (10)	0.0040 (9)	−0.0162 (10)
O8	0.0544 (13)	0.0426 (12)	0.0298 (10)	−0.0322 (11)	0.0046 (9)	−0.0139 (9)
C8	0.109 (3)	0.0298 (17)	0.041 (2)	−0.006 (2)	0.019 (2)	−0.0141 (16)
O9	0.0666 (15)	0.0410 (12)	0.0278 (10)	−0.0306 (11)	0.0047 (10)	−0.0149 (10)
C9	0.0446 (17)	0.0289 (15)	0.0403 (16)	−0.0114 (13)	−0.0100 (14)	−0.0121 (13)
C10	0.090 (3)	0.047 (2)	0.077 (3)	−0.030 (2)	−0.052 (2)	0.001 (2)
O10	0.0417 (11)	0.0367 (11)	0.0298 (10)	−0.0172 (10)	0.0012 (9)	−0.0158 (10)
C11	0.0308 (14)	0.0283 (14)	0.0371 (15)	−0.0113 (12)	−0.0021 (12)	−0.0120 (13)
C12	0.0366 (18)	0.066 (2)	0.051 (2)	0.0004 (17)	−0.0074 (16)	−0.0263 (19)
C13	0.0335 (14)	0.0277 (14)	0.0294 (14)	−0.0152 (12)	0.0017 (11)	−0.0094 (12)
C14	0.096 (3)	0.062 (2)	0.048 (2)	−0.061 (2)	0.019 (2)	−0.0251 (18)

### catena-Poly[[manganese(II)]-µ3-acetato-µ2-acetato-µ-(2-methylpyridine N-oxide)-[aquamanganese(II)]-di-µ2-acetato-manganese(II)-di-µ2-acetato-µ3-acetato-[aquamanganese(II)]-µ2-acetato-µ-(2-methylpyridine N-oxide)] (II) Geometric parameters (Å, º)

**Table:**

Mn1—O1	2.2061 (19)	C4—C5	1.374 (4)
Mn1—O1^i^	2.2061 (19)	O4—C9	1.244 (4)
Mn1—O2^i^	2.159 (2)	O5—C9	1.263 (3)
Mn1—O2	2.159 (2)	C5—H5	0.9500
Mn1—O4	2.129 (2)	C6—H6A	0.9800
Mn1—O4^i^	2.129 (2)	C6—H6B	0.9800
C1—N1	1.354 (4)	C6—H6C	0.9800
C1—C2	1.389 (4)	O6—C11	1.246 (3)
C1—C6	1.491 (4)	C7—C8	1.495 (4)
O1—N1	1.359 (3)	O7—C11	1.253 (3)
O1—Mn2	2.2300 (18)	O8—C13	1.258 (3)
N1—C5	1.346 (4)	C8—H8A	0.9800
C2—H2	0.9500	C8—H8C	0.9800
C2—C3	1.385 (5)	C8—H8B	0.9800
O2—C7	1.232 (3)	O9—C13	1.247 (3)
Mn2—O3^i^	2.2224 (18)	C9—C10	1.513 (4)
Mn2—O5	2.177 (2)	C10—H10A	0.9800
Mn2—O6	2.136 (2)	C10—H10B	0.9800
Mn2—O8	2.182 (2)	C10—H10C	0.9800
Mn2—O10	2.239 (2)	O10—H10D	0.838 (18)
C3—H3	0.9500	O10—H10E	0.846 (18)
C3—C4	1.379 (5)	C11—C12	1.513 (4)
O3—Mn3^ii^	2.3091 (18)	C12—H12B	0.9800
O3—C7	1.286 (3)	C12—H12C	0.9800
Mn3—O7^iii^	2.157 (2)	C12—H12A	0.9800
Mn3—O7	2.157 (2)	C13—C14	1.511 (4)
Mn3—O9	2.1453 (19)	C14—H14A	0.9800
Mn3—O9^iii^	2.1452 (19)	C14—H14C	0.9800
C4—H4	0.9500	C14—H14B	0.9800
			
O1^i^—Mn1—O1	180.0	O9—Mn3—O7	93.91 (8)
O2^i^—Mn1—O1	85.03 (7)	O9^iii^—Mn3—O7^iii^	93.91 (8)
O2—Mn1—O1^i^	85.03 (7)	O9^iii^—Mn3—O9	180.0
O2—Mn1—O1	94.97 (7)	C3—C4—H4	120.1
O2^i^—Mn1—O1^i^	94.97 (7)	C5—C4—C3	119.8 (3)
O2—Mn1—O2^i^	180.0	C5—C4—H4	120.1
O4^i^—Mn1—O1^i^	91.37 (8)	C9—O4—Mn1	132.59 (19)
O4—Mn1—O1	91.37 (8)	C9—O5—Mn2	133.4 (2)
O4—Mn1—O1^i^	88.63 (8)	N1—C5—C4	119.8 (3)
O4^i^—Mn1—O1	88.63 (8)	N1—C5—H5	120.1
O4^i^—Mn1—O2^i^	91.45 (8)	C4—C5—H5	120.1
O4^i^—Mn1—O2	88.55 (8)	C1—C6—H6A	109.5
O4—Mn1—O2	91.45 (8)	C1—C6—H6B	109.5
O4—Mn1—O2^i^	88.55 (8)	C1—C6—H6C	109.5
O4—Mn1—O4^i^	180.0	H6A—C6—H6B	109.5
N1—C1—C2	117.6 (3)	H6A—C6—H6C	109.5
N1—C1—C6	117.2 (3)	H6B—C6—H6C	109.5
C2—C1—C6	125.2 (3)	C11—O6—Mn2	136.82 (19)
Mn1—O1—Mn2	110.48 (7)	O2—C7—O3	123.2 (2)
N1—O1—Mn1	120.93 (14)	O2—C7—C8	117.5 (2)
N1—O1—Mn2	119.74 (14)	O3—C7—C8	119.4 (2)
C1—N1—O1	118.8 (2)	C11—O7—Mn3	134.16 (18)
C5—N1—C1	122.8 (3)	C13—O8—Mn2	134.65 (18)
C5—N1—O1	118.4 (2)	C7—C8—H8A	109.5
C1—C2—H2	119.5	C7—C8—H8C	109.5
C3—C2—C1	121.0 (3)	C7—C8—H8B	109.5
C3—C2—H2	119.5	H8A—C8—H8C	109.5
C7—O2—Mn1	140.53 (19)	H8A—C8—H8B	109.5
O1—Mn2—O10	88.73 (7)	H8C—C8—H8B	109.5
O3^i^—Mn2—O1	89.31 (7)	C13—O9—Mn3	139.90 (18)
O3^i^—Mn2—O10	176.15 (7)	O4—C9—O5	125.4 (3)
O5—Mn2—O1	97.41 (7)	O4—C9—C10	118.2 (3)
O5—Mn2—O3^i^	101.91 (7)	O5—C9—C10	116.4 (3)
O5—Mn2—O8	87.26 (8)	C9—C10—H10A	109.5
O5—Mn2—O10	81.63 (8)	C9—C10—H10B	109.5
O6—Mn2—O1	86.79 (8)	C9—C10—H10C	109.5
O6—Mn2—O3^i^	97.97 (7)	H10A—C10—H10B	109.5
O6—Mn2—O5	159.71 (8)	H10A—C10—H10C	109.5
O6—Mn2—O8	88.10 (8)	H10B—C10—H10C	109.5
O6—Mn2—O10	78.61 (8)	Mn2—O10—H10D	112 (2)
O8—Mn2—O1	174.87 (7)	Mn2—O10—H10E	115 (2)
O8—Mn2—O3^i^	91.81 (7)	H10D—O10—H10E	99 (3)
O8—Mn2—O10	89.86 (8)	O6—C11—O7	125.7 (3)
C2—C3—H3	120.6	O6—C11—C12	116.1 (3)
C4—C3—C2	118.8 (3)	O7—C11—C12	118.3 (3)
C4—C3—H3	120.6	C11—C12—H12B	109.5
Mn2^i^—O3—Mn3^ii^	110.16 (7)	C11—C12—H12C	109.5
C7—O3—Mn2^i^	117.09 (16)	C11—C12—H12A	109.5
C7—O3—Mn3^ii^	132.72 (17)	H12B—C12—H12C	109.5
O3^iv^—Mn3—O3^i^	180.0	H12B—C12—H12A	109.5
O7^iii^—Mn3—O3^i^	88.03 (7)	H12C—C12—H12A	109.5
O7—Mn3—O3^iv^	88.03 (7)	O8—C13—C14	117.4 (2)
O7—Mn3—O3^i^	91.97 (7)	O9—C13—O8	125.2 (3)
O7^iii^—Mn3—O3^iv^	91.97 (7)	O9—C13—C14	117.3 (2)
O7—Mn3—O7^iii^	180.0	C13—C14—H14A	109.5
O9^iii^—Mn3—O3^i^	88.85 (7)	C13—C14—H14C	109.5
O9^iii^—Mn3—O3^iv^	91.15 (7)	C13—C14—H14B	109.5
O9—Mn3—O3^iv^	88.85 (7)	H14A—C14—H14C	109.5
O9—Mn3—O3^i^	91.15 (7)	H14A—C14—H14B	109.5
O9^iii^—Mn3—O7	86.09 (8)	H14C—C14—H14B	109.5
O9—Mn3—O7^iii^	86.09 (8)		

Symmetry codes: (i) −*x*+1, −*y*+1, −*z*+1; (ii) *x*, *y*+1, *z*−1; (iii) −*x*+1, −*y*, −*z*+2; (iv) *x*, *y*−1, *z*+1.

### catena-Poly[[manganese(II)]-µ3-acetato-µ2-acetato-µ-(2-methylpyridine N-oxide)-[aquamanganese(II)]-di-µ2-acetato-manganese(II)-di-µ2-acetato-µ3-acetato-[aquamanganese(II)]-µ2-acetato-µ-(2-methylpyridine N-oxide)] (II) Hydrogen-bond geometry (Å, º)

**Table:**

*D*—H···*A*	*D*—H	H···*A*	*D*···*A*	*D*—H···*A*
O10—H10*D*···O5^v^	0.84 (2)	2.04 (2)	2.821 (3)	155 (3)
O10—H10*E*···O8^v^	0.85 (2)	1.94 (2)	2.727 (3)	155 (3)

Symmetry code: (v) −*x*+1, −*y*, −*z*+1.

### catena-Poly[[manganese(II)-di-µ2-acetato-µ-(4-methylpyridine N-oxide)] monohydrate] (III) Crystal data

**Table:**

[Mn(C_2_H_3_O_2_)_2_(C_6_H_7_NO)]·H_2_O	*F*(000) = 620
*M**_r_* = 300.17	*D*_x_ = 1.542 Mg m^−^^3^
Monoclinic, *P*2_1_/*n*	Mo *K*α radiation, λ = 0.71073 Å
*a* = 11.100 (3) Å	Cell parameters from 2982 reflections
*b* = 7.334 (3) Å	θ = 2.1–27.5°
*c* = 15.9808 (4) Å	µ = 1.04 mm^−^^1^
β = 96.500 (11)°	*T* = 173 K
*V* = 1292.6 (6) Å^3^	Prism, colorless
*Z* = 4	0.2 × 0.05 × 0.05 mm

### catena-Poly[[manganese(II)-di-µ2-acetato-µ-(4-methylpyridine N-oxide)] monohydrate] (III) Data collection

**Table:**

Rigaku XtaLAB mini diffractometer	2224 reflections with *I* > 2σ(*I*)
Detector resolution: 13.6612 pixels mm^-1^	*R*_int_ = 0.074
profile data from ω–scans	θ_max_ = 27.6°, θ_min_ = 2.1°
Absorption correction: multi-scan (*REQAB*; Rigaku, 1998)	*h* = −14→14
*T*_min_ = 0.850, *T*_max_ = 1.00	*k* = −9→9
13236 measured reflections	*l* = −20→20
2957 independent reflections	

### catena-Poly[[manganese(II)-di-µ2-acetato-µ-(4-methylpyridine N-oxide)] monohydrate] (III) Refinement

**Table:**

Refinement on *F*^2^	Hydrogen site location: mixed
Least-squares matrix: full	H atoms treated by a mixture of independent and constrained refinement
*R*[*F*^2^ > 2σ(*F*^2^)] = 0.045	*w* = 1/[σ^2^(*F*_o_^2^) + (0.0269*P*)^2^ + 1.0998*P*] where *P* = (*F*_o_^2^ + 2*F*_c_^2^)/3
*wR*(*F*^2^) = 0.105	(Δ/σ)_max_ = 0.001
*S* = 1.07	Δρ_max_ = 0.35 e Å^−^^3^
2957 reflections	Δρ_min_ = −0.40 e Å^−^^3^
173 parameters	Extinction correction: SHELXL-2018/1 (Sheldrick 2015), Fc^*^=kFc[1+0.001xFc^2^λ^3^/sin(2θ)]^-1/4^
2 restraints	Extinction coefficient: 0.0042 (9)

### catena-Poly[[manganese(II)-di-µ2-acetato-µ-(4-methylpyridine N-oxide)] monohydrate] (III) Special details

**Table:**

Geometry. All esds (except the esd in the dihedral angle between two l.s. planes) are estimated using the full covariance matrix. The cell esds are taken into account individually in the estimation of esds in distances, angles and torsion angles; correlations between esds in cell parameters are only used when they are defined by crystal symmetry. An approximate (isotropic) treatment of cell esds is used for estimating esds involving l.s. planes.

### catena-Poly[[manganese(II)-di-µ2-acetato-µ-(4-methylpyridine N-oxide)] monohydrate] (III) Fractional atomic coordinates and isotropic or equivalent isotropic displacement parameters (Å^2^)

**Table:**

	*x*	*y*	*z*	*U*_iso_*/*U*_eq_	
Mn1	0.25529 (4)	0.54319 (6)	0.75049 (3)	0.02337 (15)	
O1	0.35635 (15)	0.7980 (2)	0.73689 (12)	0.0243 (4)	
C1	0.5439 (2)	0.8534 (4)	0.69051 (19)	0.0316 (7)	
H1	0.505841	0.869094	0.636076	0.038*	
N1	0.47864 (19)	0.8127 (3)	0.75356 (14)	0.0235 (5)	
C2	0.6685 (3)	0.8721 (4)	0.7071 (2)	0.0354 (7)	
H2	0.713428	0.902878	0.663478	0.042*	
O2	0.33022 (19)	0.4486 (3)	0.63859 (13)	0.0372 (5)	
O3	0.3188 (2)	0.1450 (3)	0.63793 (13)	0.0389 (5)	
C3	0.7278 (2)	0.8459 (4)	0.7874 (2)	0.0323 (7)	
C4	0.6557 (3)	0.8040 (4)	0.85053 (19)	0.0321 (7)	
H4	0.691779	0.786075	0.905330	0.039*	
O4	0.10483 (18)	0.6385 (3)	0.66703 (14)	0.0402 (6)	
O5	0.09920 (19)	0.9411 (3)	0.66333 (14)	0.0385 (5)	
C5	0.5315 (3)	0.7885 (4)	0.83299 (18)	0.0284 (6)	
H5	0.484307	0.761524	0.875895	0.034*	
C6	0.8641 (3)	0.8641 (5)	0.8053 (2)	0.0455 (8)	
H6A	0.884813	0.903431	0.862398	0.068*	
H6B	0.892537	0.952060	0.767630	0.068*	
H6C	0.901330	0.748229	0.797218	0.068*	
C7	0.3391 (2)	0.2954 (4)	0.60495 (17)	0.0259 (6)	
C8	0.3785 (3)	0.2898 (5)	0.51770 (19)	0.0379 (7)	
H8A	0.351789	0.398675	0.487760	0.057*	
H8B	0.343341	0.185322	0.488106	0.057*	
H8C	0.465247	0.282015	0.521701	0.057*	
C9	0.0622 (2)	0.7859 (4)	0.63902 (18)	0.0271 (6)	
C10	−0.0435 (3)	0.7767 (5)	0.5705 (2)	0.0461 (9)	
H10A	−0.083947	0.892610	0.565920	0.069*	
H10B	−0.099334	0.684427	0.584238	0.069*	
H10C	−0.014279	0.747388	0.517831	0.069*	
O6	0.3091 (2)	0.7951 (4)	0.53409 (15)	0.0440 (6)	
H6D	0.301 (4)	0.882 (4)	0.567 (2)	0.066*	
H6E	0.313 (4)	0.708 (4)	0.568 (2)	0.066*	

### catena-Poly[[manganese(II)-di-µ2-acetato-µ-(4-methylpyridine N-oxide)] monohydrate] (III) Atomic displacement parameters (Å^2^)

**Table:**

	*U*^11^	*U*^22^	*U*^33^	*U*^12^	*U*^13^	*U*^23^
Mn1	0.0222 (2)	0.0195 (2)	0.0284 (2)	−0.00149 (16)	0.00291 (17)	−0.00006 (16)
O1	0.0159 (9)	0.0210 (10)	0.0355 (11)	−0.0003 (7)	0.0009 (8)	−0.0011 (8)
C1	0.0253 (14)	0.0377 (18)	0.0317 (16)	−0.0018 (13)	0.0033 (12)	0.0015 (13)
N1	0.0167 (10)	0.0223 (12)	0.0311 (12)	−0.0014 (9)	0.0014 (9)	−0.0015 (10)
C2	0.0279 (15)	0.0411 (19)	0.0388 (17)	−0.0032 (14)	0.0105 (13)	−0.0054 (15)
O2	0.0438 (13)	0.0300 (12)	0.0403 (13)	−0.0022 (10)	0.0155 (10)	−0.0046 (9)
O3	0.0518 (13)	0.0287 (12)	0.0396 (12)	0.0006 (10)	0.0192 (11)	0.0044 (10)
C3	0.0227 (14)	0.0291 (17)	0.0445 (18)	0.0001 (12)	0.0006 (13)	−0.0065 (13)
C4	0.0298 (15)	0.0320 (17)	0.0327 (16)	0.0002 (12)	−0.0047 (13)	−0.0015 (13)
O4	0.0329 (11)	0.0293 (12)	0.0547 (14)	−0.0023 (9)	−0.0114 (10)	0.0081 (10)
O5	0.0343 (12)	0.0250 (12)	0.0529 (14)	0.0027 (9)	−0.0093 (10)	−0.0067 (10)
C5	0.0283 (15)	0.0279 (16)	0.0289 (15)	0.0011 (12)	0.0031 (12)	−0.0002 (12)
C6	0.0270 (16)	0.047 (2)	0.061 (2)	−0.0010 (15)	0.0000 (15)	−0.0082 (18)
C7	0.0215 (13)	0.0306 (16)	0.0259 (14)	0.0000 (11)	0.0036 (11)	−0.0001 (12)
C8	0.0426 (18)	0.0417 (19)	0.0307 (16)	−0.0055 (14)	0.0097 (14)	0.0014 (14)
C9	0.0221 (14)	0.0310 (16)	0.0284 (15)	0.0022 (12)	0.0033 (12)	0.0028 (12)
C10	0.043 (2)	0.043 (2)	0.047 (2)	−0.0048 (16)	−0.0164 (17)	0.0023 (16)
O6	0.0536 (15)	0.0473 (16)	0.0306 (12)	−0.0011 (13)	0.0023 (11)	−0.0039 (10)

### catena-Poly[[manganese(II)-di-µ2-acetato-µ-(4-methylpyridine N-oxide)] monohydrate] (III) Geometric parameters (Å, º)

**Table:**

Mn1—O1^i^	2.206 (2)	C4—C5	1.380 (4)
Mn1—O1	2.203 (2)	O4—C9	1.243 (3)
Mn1—O2	2.170 (2)	O5—C9	1.257 (3)
Mn1—O3^ii^	2.179 (2)	C5—H5	0.9300
Mn1—O4	2.134 (2)	C6—H6A	0.9600
Mn1—O5^i^	2.136 (2)	C6—H6B	0.9600
O1—N1	1.358 (3)	C6—H6C	0.9600
C1—H1	0.9300	C7—C8	1.508 (4)
C1—N1	1.339 (4)	C8—H8A	0.9600
C1—C2	1.386 (4)	C8—H8B	0.9600
N1—C5	1.348 (4)	C8—H8C	0.9600
C2—H2	0.9300	C9—C10	1.513 (4)
C2—C3	1.387 (4)	C10—H10A	0.9600
O2—C7	1.254 (3)	C10—H10B	0.9600
O3—C7	1.254 (3)	C10—H10C	0.9600
C3—C4	1.391 (4)	O6—H6D	0.841 (19)
C3—C6	1.514 (4)	O6—H6E	0.839 (18)
C4—H4	0.9300		
			
O1—Mn1—O1^i^	176.46 (6)	C5—C4—C3	120.9 (3)
O2—Mn1—O1^i^	94.96 (8)	C5—C4—H4	119.5
O2—Mn1—O1	86.72 (8)	C9—O4—Mn1	138.5 (2)
O2—Mn1—O3^ii^	178.58 (8)	C9—O5—Mn1^ii^	135.55 (19)
O3^ii^—Mn1—O1^i^	86.37 (8)	N1—C5—C4	119.9 (3)
O3^ii^—Mn1—O1	91.92 (8)	N1—C5—H5	120.0
O4—Mn1—O1	91.80 (8)	C4—C5—H5	120.0
O4—Mn1—O1^i^	85.20 (8)	C3—C6—H6A	109.5
O4—Mn1—O2	86.30 (9)	C3—C6—H6B	109.5
O4—Mn1—O3^ii^	93.32 (9)	C3—C6—H6C	109.5
O4—Mn1—O5^i^	177.62 (9)	H6A—C6—H6B	109.5
O5^i^—Mn1—O1	90.27 (8)	H6A—C6—H6C	109.5
O5^i^—Mn1—O1^i^	92.69 (8)	H6B—C6—H6C	109.5
O5^i^—Mn1—O2	94.99 (9)	O2—C7—C8	117.7 (3)
O5^i^—Mn1—O3^ii^	85.44 (9)	O3—C7—O2	125.5 (3)
Mn1—O1—Mn1^ii^	112.64 (8)	O3—C7—C8	116.7 (3)
N1—O1—Mn1	123.84 (15)	C7—C8—H8A	109.5
N1—O1—Mn1^ii^	118.48 (15)	C7—C8—H8B	109.5
N1—C1—H1	120.3	C7—C8—H8C	109.5
N1—C1—C2	119.4 (3)	H8A—C8—H8B	109.5
C2—C1—H1	120.3	H8A—C8—H8C	109.5
C1—N1—O1	118.9 (2)	H8B—C8—H8C	109.5
C1—N1—C5	121.5 (2)	O4—C9—O5	125.3 (3)
C5—N1—O1	119.5 (2)	O4—C9—C10	117.0 (3)
C1—C2—H2	119.3	O5—C9—C10	117.6 (3)
C1—C2—C3	121.5 (3)	C9—C10—H10A	109.5
C3—C2—H2	119.3	C9—C10—H10B	109.5
C7—O2—Mn1	134.09 (19)	C9—C10—H10C	109.5
C7—O3—Mn1^i^	138.4 (2)	H10A—C10—H10B	109.5
C2—C3—C4	116.8 (3)	H10A—C10—H10C	109.5
C2—C3—C6	121.5 (3)	H10B—C10—H10C	109.5
C4—C3—C6	121.8 (3)	H6D—O6—H6E	100 (4)
C3—C4—H4	119.5		

Symmetry codes: (i) −*x*+1/2, *y*−1/2, −*z*+3/2; (ii) −*x*+1/2, *y*+1/2, −*z*+3/2.

### catena-Poly[[manganese(II)-di-µ2-acetato-µ-(4-methylpyridine N-oxide)] monohydrate] (III) Hydrogen-bond geometry (Å, º)

**Table:**

*D*—H···*A*	*D*—H	H···*A*	*D*···*A*	*D*—H···*A*
O6—H6*D*···O3^iii^	0.84 (2)	2.23 (2)	3.052 (3)	165 (4)
O6—H6*E*···O2	0.84 (2)	2.21 (2)	3.035 (3)	170 (4)

Symmetry code: (iii) *x*, *y*+1, *z*.

## References

[bb1] Ciunik, Z. & Głowiak, T. (1980). *Acta Cryst.* B**36**, 2029–2033.

[bb2] Dave, B. C., Czernuszewicz, R. S., Bond, M. R. & Carrano, C. J. (1993). *Inorg. Chem.* **32**, 3593–3594.

[bb3] Dolomanov, O. V., Bourhis, L. J., Gildea, R. J., Howard, J. A. K. & Puschmann, H. (2009). *J. Appl. Cryst.* **42**, 339–341.

[bb4] Groom, C. R., Bruno, I. J., Lightfoot, M. P. & Ward, S. C. (2016). *Acta Cryst.* B**72**, 171–179. 10.1107/S2052520616003954 PMC4822653 27048719

[bb5] Kang, L., Lynch, G., Lynch, W. & Padgett, C. (2017). *Acta Cryst.* E**73**, 1434–1438. 10.1107/S2056989017012038 PMC5730290 29250353

[bb6] Liu, J., Chen, L., Cui, H., Zhang, Y., Zhang, L. & Su, C.-Y. (2014). *Chem. Soc. Rev.* **43**, 6011–6061. 10.1039/c4cs00094c 24871268

[bb7] Mondal, S., Guha, A., Suresh, E., Jana, A. D. & Banerjee, A. (2012). *J. Mol. Struct.* **1029**, 169–174.

[bb8] Ren, X.-H., Wang, P., Cheng, J.-Y. & Dong, Y.-B. (2018). *J. Mol. Struct.* **1161**, 145–151.

[bb9] Rigaku (1998). *REQAB*. Rigaku Corporation, Tokyo, Japan.

[bb10] Rigaku (2009). *CrystalClear*. Rigaku Corporation, Tokyo, Japan.

[bb11] Sarma, R. & Baruah, J. B. (2011). *Solid State Sci.* **13**, 1692–1700.

[bb12] Sarma, R., Karmakar, A. & Baruah, J. B. (2008). *Inorg. Chim. Acta*, **361**, 2081–2086.

[bb13] Sarma, R., Perumal, A. & Baruah, J. B. (2009). *J. Coord. Chem.* **62**, 1513–1524.

[bb14] Sheldrick, G. M. (2015*a*). *Acta Cryst.* A**71**, 3–8.

[bb16] Sheldrick, G. M. (2015*b*). *Acta Cryst.* C**71**, 3–8.

[bb15] Sniekers, J., Malaquias, J. C., Van Meervelt, L., Fransaer, J. & Binnemans, K. (2017). *Dalton Trans.* **46**, 2497–2509. 10.1039/c6dt04781e 28144661

